# Electrophysiological Evidence for Domain-General Inhibitory Control during Bilingual Language Switching

**DOI:** 10.1371/journal.pone.0110887

**Published:** 2014-10-24

**Authors:** Huanhuan Liu, Sonja Rossi, Huixia Zhou, Baoguo Chen

**Affiliations:** 1 School of Psychology, Beijing Normal University, Beijing, China; 2 Department of Medical Psychology, Innsbruck Medical University, Innsbruck, Austria; 3 Department of Neurology, Max Planck Institute for Human Cognitive and Brain Sciences, Leipzig, Germany; University of Western Ontario, Canada

## Abstract

This paper presents an experiment that explored the role of domain–general inhibitory control on language switching. Reaction times (RTs) and event–related brain potentials (ERPs) were recorded when low–proficient bilinguals with high and low inhibitory control (IC) switched between overt picture naming in both their L1 and L2. Results showed that the language switch costs of bilinguals with high–IC were symmetrical, while that of bilinguals with low–IC were not. The N2 component failed to show a significant interaction between group, language and task, indicating that inhibition may not comes into play during the language task schema competition phase. The late positive component (LPC), however, showed larger amplitudes for L2 repeat and switch trials than for L1 trials in the high–IC group, indicating that inhibition may play a key role during the lexical response selection phase. These findings suggest that domain–general inhibitory control plays an important role in modulating language switch costs and its influence can be specified in lexical selection phase.

## Introduction

Switching between languages can sometimes cause processing delays, which is referred to as the so–called “switch costs”. Switch costs can be symmetrical, meaning that comparable switch costs occur when switching from the more dominant language (L1) to the less dominant language (L2) or vice versa. However, in some cases longer processing times were observed when switching to the more dominant language (L2 to L1) than to the less dominant language (L1 to L2), which thus leads to asymmetrical language switch costs [1]–[3]. Many studies found that symmetrical or asymmetrical language switch costs might not only be of a linguistic nature but are also influenced by other factors such as the necessary degree of inhibitory control [1]–[5].

An important account in language switching research is the inhibitory control model (IC model) [4]. The model argued that the degree of inhibition for language is positively correlated with language proficiency and the reactivation of a suppressed language is positively correlated with the degree of inhibition. This means that a dominant language needs to recruit a more laborious inhibition and reactivation. More specifically, due to the high inhibition needed to suppress a dominant L1 in the previous trial when switching from L1 to L2, participants spend more time in relieving the inhibition when switching from L2 to L1, thus leading to larger switch costs from L2 to L1 than vice versa. In contrast, if participants could have balanced degrees of inhibition and disinhibition, they may be expected to exhibit similar switch costs between the more dominant (L1) and the less dominant language (L2). Evidences from behavioral experiments have been taken as support for the IC model [1]–[3], [6].

However, behavioral approaches do not allow tracking the fine–grained timing mechanisms of inhibition acting during language switching. According to Green's [4] IC model, inhibition may occur at two possible phases, that is, the language task schema competition phase, where the language task schema competes with each other (i.e., while selecting a more dominant or a less dominant language), and the lexical response selection phase, where the winning target language task schema inhibits lemmas of the non–target language. The event related potential (ERP) technique bears the great potential of online tracking the time course of cognitive processing, so evidence from electrophysiological studies may provide important information about when and how inhibition interacts with language switching.

It is well known that language switching is associated with a significant increase in amplitude of the frontal N2 component. Some studies suggest that the N2 reflects inhibitory control mechanism [5], [7], and inhibition might come into play during the language task schema competition phase. For example, Jackson et al. [5] required low-proficient bilinguals to perform a speeded digit naming task in their L1 and L2 according to a visual cue (the color of the digit). The N2 component, which was recorded over the fronto–central region of the scalp, was significantly enhanced in amplitude (i.e., more negative) in L2 switch trials (L1 to L2) compared with L1 switch trials (L2 to L1). This modulatory pattern of the N2 seems to be in line with the IC model, that is, switching from the dominant language to the weak language needs more inhibition of the dominant language, displaying larger N2 amplitudes. Thus, the authors conclude that inhibition occurs during the language task schema competition phase. Additionally, they found that both L1 and L2 elicited larger amplitudes with respect to the sustained response of the LPC component for switch trials compared to non–switch trials. They speculated that the LPC found in switch trials may reflect a different form of inhibitory control than that reflected by the frontal N2. Specifically, the LPC is assumed to represent disinhibition between L1 and L2, which is needed to reactivate the access to the previously suppressed lexical item.

However, some researchers questioned the involvement of inhibitory control during language switching. They argued that the N2 more likely reflects attentional control mechanisms rather that inhibitory processes. For example, Verhoef and colleagues [8] explored preparation effects during an L1 and L2 picture naming task in low–proficient Dutch–English bilinguals. The behavioral results showed asymmetrical switch costs on short–cue stimuli intervals (500 ms) and symmetrical costs on long–cue stimuli intervals (1250 ms), but picture naming in L1 and L2 failed to show larger N2 effects for switch trials compared to repeat trials. Thus, their study does not support the predictions derived from the IC model. Additionally, they found that preparation interval effects were selectively absent for the L1 repeat trials in both RT and N2 data. So they assumed that larger switch costs for L1 compared to L2 arise from the fact that L1 repeat trials display disproportionately fast reactions. For low–proficient bilinguals, in all conditions except L1 repeat trials, switching between L1 and L2 suffers from competition from the non–target language, resulting in relatively fast L1 repeat responses and thus asymmetrical switch costs. In other words, preparation time (short versus long cue–stimulus intervals) modulated competition, however inhibition was not necessary for language switching. Verhoef et al. [9] further confirmed this view in a subsequent study. They observed the N2 (200–350 ms) being more negative in switch trials compared to repeat trials for L2, but not for L1. Thus, their results confirm that relatively fast L1 repeat responses lead to asymmetrical switch costs suggesting that the N2 reflects attentional control rather than inhibition.

Previous studies on domain-general inhibitory control considered attentional control as part of the stimulus–driven attentional system [10], [11], which is involved in the bottom–up control of attention. Thus, some studies interpreted the N2 as a reflection of attentional control or cognitive control during the preparation phase, and they also assume that the N2 refers to representations of cueing information about the to–be–performed task [10]–[13]. In other words, attentional control is responsible for controlling and monitoring appropriate responses in order to subsequently allow stimulus evaluation to be completed during the response phase [14].

Recently, some researchers proposed that inhibition indeed plays a crucial role during language switching but that it occurs at the later lexical response selection phase reflected by the late positive component (LPC) in the EEG [15]. Martin et al. [15] used a picture naming task to explore the language control mechanisms of early and late bilinguals. Early bilinguals were subdivided into two groups: (1) Spanish (L1) – Catalan (L2) early bilinguals who learned English (L3) late in time and who performed the picture naming task in L1 and L3 (this group was termed L1L3 early bilinguals) and (2) Spanish–Catalan early bilinguals who performed the task in L1 and L2 (termed L1L2 early bilinguals). Additionally, late bilinguals also performed the task in L1 and L3 (termed L1L3 late bilinguals). It should be noted that the L3 was always considered as the weakest language. The N2 component was larger for early than that for late L1L3 bilinguals, but this component showed a similar pattern for L1L3 and L1L2 early bilinguals. Thus, they proposed that the N2 does not reflect inhibition per se, however, it resembles processes involved in cognitive control. Furthermore, they found that the LPC was larger in L1L2 early bilinguals than L1L3 early bilinguals, as well as larger in switch than in non-switch trials when responses were given in L3. The authors suggested that the LPC is related to the consequences of inhibition at the level of specific lexical response selection.

Some fMRI (functional magnetic resonance imaging) studies also observed that inhibitory control plays a key role in language switching, and the inhibitory control mechanism activate the right inferior frontal gyrus (rIFG) which is considered a domain-general inhibitory control area [2], [16]–[19]. For example, De Bruin et al. [2] found that the right inferior frontal gyrus (rIFG) and the pre–supplementary motor area (pre–SMA) were both involved in switching to L2 and L3. Thus, they concluded that trilinguals use domain–general inhibition to switch among their languages. It seems that domain-specific inhibition underlies the language network and plays a parallel role in language switching.

Considering the experimental evidences so far, it is not clear whether domain- general inhibition is necessary during language switching and whether domain– general inhibition underlies language switching mechanisms. If domain– general inhibition plays the predominant role in modulating language switch costs, a further question arises, namely, at what timing these mechanisms come into play. The present study aims at exploring the role of domain-general inhibitory control during language switching, specifically, when in time these inhibitory processes are active. In order to track these fine–grained timing issues, ERPs will be adopted as the method of choice.

In order to offset the impact of L2 proficiency on language switching, we selected low– proficient Chinese– English bilinguals divided into two groups (i.e., high inhibitory control – high-IC – group vs. low inhibitory control – low-IC – group) based on a domain-general inhibitory control task (a modified Simon task). Both groups took part in a language switching task. If domain-general inhibition can be generalized to language switching as proposed by the IC model, we predict symmetrical language switch costs for the high–IC group and asymmetrical costs for the low–IC group observable in the behavioral results. Electrophysiological data, however, will provide further specification about the underlying timing mechanisms. According to the IC model, since L2 switch trials will require more inhibition to suppress the strong interference from L1, these are expected to elicit a larger N2 or LPC than L1 switch trials. Specifically, if the high–IC group will show a larger N2 component in L2 switching trials than L1 trials during an early time window, while the low–IC group will not, we assume that inhibition occurs during the language task schema competition phase. If the high–IC group displays a larger LPC in L2 switching trials than L1 trials, while the low–IC group does not, this will suggest that inhibition occurs during the later lexical response selection phase. Note, that a larger N2 or LPC in L2 switch trials is assumed to suggest that participants can well inhibit the interference from the non–target language online [5], [8], [9].

## Method

### Participants

#### Demographic and language proficiency data

Forty–seven students took part in the study. They were all undergraduate students from Beijing Normal University. They were paid for participation. The study was approved by ethics committee of School of Psychology, Beijing Normal University. All participants signed the written informed consent. All participants were right handed native Chinese speakers with normal or corrected–to–normal vision, who learned English as a second language starting around the age of 8. None of the participants had neurological or psychological impairments or had used psychoactive medication. Data from five participants were eliminated, two due to behavioral reasons (too many errors) and three due to excessive EEG artifacts. The final sample consisted of 42 participants, 29 female, aged from 17 to 24 years (*M* = 21.98±1.99 years). A self-rating questionnaire was used to obtain proficiency scores. Participants needed to indicate how well their L2 (English) skills (listening, speaking, reading, and writing) were compared to L1 (Chinese). The scores were on a five point scale, in which 5 indicated that L2 skills corresponded to a native level and 1 indicated very low L2 skills, much lower than L1 skills. The average proficiency of L1 listening, speaking, reading, and writing of all participants was 4.55±.50, 4.50±.51, 4.43±.50, 4.43±.50, and of L2 2.19±.59, 2.24±.69, 2.48±.55, 2.55±.50. Paired samples *t*-tests revealed a statistically significant difference between the proficiency ratings of L1 and L2 (*t* (41) = 22.07, *p*<.001, *t* (41) = 17.70, *p*<.001, *t*(41) = 15.92, *p*<.001, *t* (41) = 20.57, *p*<.001). These results demonstrate that subjects were low proficient Chinese-English bilinguals. Furthermore, by means of the Simon task assessing inhibitory control (IC), participants were subdivided into high-IC and low-IC participants (for a description on the subdivision procedure, please refer to the next section). The average proficiency of L1 listening, speaking, reading, and writing of the high-IC group was 4.48±.51, 4.33±.48, 4.48±.51, 4.43±.51, and of the low-IC group 4.62±.50, 4.47±.48, 4.38±.50, 4.43±.51. Independent samples *t*-tests showed no significant difference between high and low-IC participants on the above mentioned four ratings (*t* (40) = −.92, *p*>.05, *t* (40) = −.61, *p*>.05, *t* (40) = .61, *p*>.05, *t* (40) = .00, *p* = 1.00). The average proficiency of L2 listening, speaking, reading, and writing of the high-IC group was 2.10±.54, 2.33±.66, 2.48±.51, 2.67±.48, and of the low-IC group 2.29±.64, 2.14±.73, 2.48±.60, 2.43±.51. Again, no significant difference between the groups was present (*t* (40) = −1.04, *p*>.05, *t* (40) = .89, *p*>.05, *t* (40) = .00, *p* = 1.00, *t* (40) = 1.56, *p*>.05).

#### Assessment of inhibitory control ability

We selected the Simon task as our measure of domain-general inhibitory control ability since it has recently been used in a number of bilingual studies [20], [21]. By means of this task, we assigned participants either to a group of high-IC or low-IC by the median split method. The Simon task consists of various versions, such as the Simon square task [2], [20]–[22] and the Simon arrow task [20], [21]. We opted for the Simon arrow task as it is more difficult than the Simon square task because the direction of arrows is a strong interference for incongruent trials. We designed four conditions of the Simon arrow task, including congruent repeat/switch trials and incongruent repeat/switch trials (please refer to the next section for details concerning the task). Congruent repeat/switch trials are easier than incongruent repeat/switch trials, because incongruent repeat/switch trials require the inhibition of the interference from the direction of arrows in the Simon task. According to the IC model, when performing the more difficult task (incongruent repeat/switch task), more inhibition has to be recruited to suppress the easier task (congruent repeat/switch task). In contrast, when performing the easier task, more cognitive resources are needed to disinhibit such task. Consequently, switch costs tend to be larger when switching into the easier task than into the more difficult one [23]. Thus, we assume that if the inhibition and disinhibition of congruent repeat/switch tasks and incongruent repeat/switch task take comparable time, then the congruent and incongruent switch costs will become symmetrical. Similarly, if the inhibition and disinhibition of congruent repeat/switch tasks and incongruent repeat/switch tasks need different time scales, then the congruent and incongruent switch costs will become asymmetrical. Following this line of thinking, we expect the high–IC group to show symmetrical switch costs, indicating that they could well balance the inhibition and the disinhibition. Furthermore, if the low–IC group exhibits asymmetrical switch costs, this would indicate that they need more effort than the high–IC group to inhibit or disinhibit the previous trials. We arranged 5 blocks and 96 trials per block (details are described in the following procedure section). All stimuli were pseudo-randomly presented. Prior to the actual experiment, each participant was presented with 12 practice trials.

A trial started with the presentation of a cue (red or blue square) for 250 ms, followed by a blank screen for a duration of 500 ms. Then an arrow stimulus appeared on the screen for 250 ms. Afterwards, the symbol “******” appeared indicating the participants to response as accurately and quickly as possible. If participants did not respond within 2000 ms, the symbol disappeared. Finally, a blank screen with a duration of 1000 ms followed before the next trial started. When the cue was red, participants had to give a congruent spatial response by button press which corresponded to the pointing direction of the arrow (congruent task); on the contrary, when the cue was blue, participants were required to give an opposite spatial response to the pointing direction of the arrow (incongruent task). The color-response association was counterbalanced across participants.

#### Simon task latency results

Accuracy scores were not analyzed due to more than 95% correct button presses in all conditions. The RTs were calculated from the onset of the picture presentation. Congruent switch costs were calculated by subtracting RTs of congruent repeat trials from RTs of congruent switch trials. Likewise, incongruent switch costs were calculated by subtracting RTs of incongruent repeat trials from RTs of incongruent switch trials. The larger the difference between congruent and incongruent switch costs, the more asymmetrical switch costs should arise. Conversely, the smaller the difference, the more symmetrical switch costs should be. According to the median split method, these participants were divided into two groups (i.e., high-IC group vs. low-IC group). The high–IC group is expected to show symmetrical switch costs, while the low–IC group should exhibit asymmetrical switch costs. There were 21 participants (17 female) in the high–IC and 21 participants (12 female) in the low–IC group, respectively.

In order to validate our subdivision of subjects into a high– and a low–IC group and to test whether the expected pattern of symmetric and asymmetric domain-general switch costs is observed in the high– and low–IC group, respectively, we performed a two–way repeated–measures ANOVA with the between–subject factor group (high vs. low inhibitory control group) and the within–subject factor Simon switch costs (congruent vs. incongruent switch costs). The ANOVA revealed a significant main effect of Simon switch costs (*F*(1, 40) = 30.44, *MSE*  = 594, *η^2^_P_* = .43, *p*<.001) and an interaction between group and Simon switch costs (*F*(1, 40) = 27.59, *MSE*  = 594, *η^2^_P_* = .41, *p*<.001). A simple effect analysis showed that the congruent switch costs (19 ms) and incongruent switch costs (17 ms) for the high–IC group did not differ from each other (*F*(1, 20) = .17, *p*>.05), indicating that Simon switch costs were symmetrical. The switch costs for the low–IC group, however, differed significantly (*F*(1, 20) = 32.24, *p*<.001), revealing larger congruent switch costs (52 ms) than incongruent ones (−5 ms), indicating that Simon switch costs were asymmetrical in this group.

### Language switching material

A picture naming paradigm was performed for the language switching task. Stimuli consisted of 48 black–and–white line drawings with a size of 15 cm ×15 cm. These drawings were selected from the Snodgrass and Vanderwart's photo gallery standardized by Zhang and Yang [24]. Chinese (L1) names of all pictures were two-character words, and their English (L2) equivalents were either mono- or two-syllabic words with 3–6 letters. Another separate group of 40 students from Beijing Normal University, who had similar L2 proficiency as the participants of the present experiment rated the subjective familiarity of L2 and L1 names of the pictures on a 5-point scale (1 =  “very unfamiliar”, 5 =  “very familiar”). The average subjective familiarity of L2 names was 4.81±.10; that of L1 names was 4.79±.12. Thus, there was no significant difference in familiarity (*t*(47) = 1.48, *p*>.05). The average word frequency of L2 names was 104.23±128.39 [25]; that of L1 names was 77.53±114.24 [26]. Again, there was no significant difference in word frequency (*t*(47) = 1.54, *p*>.05).

### Language switching procedure

After completing the bilingual proficiency questionnaire, participants learned to get familiar with L1 and L2 picture names in order to reduce naming errors. In the formal ERP experiment, stimuli were presented at the center of a 17-inch computer screen with 1024×768 pixel resolution. A trial started with the presentation of a cue for 250 ms. A red cue indicated to name the picture in L1, and a blue cue referred to naming the picture in L2. The color-language association was counterbalanced across participants, but all pictures had to be named in both languages. After the cues, a blank screen for a duration of 500 ms appeared. Then a picture stimulus was presented on the screen for 250 ms, followed by a blank screen for 1000 ms. Afterwards, the symbol “******” appeared indicating the participants to name the pictures as accurately and quickly as possible. Subjects were instructed to wait until the symbol appeared even in case they knew the response earlier. The delay should avoid contamination of the relevant EEG signal with myoelectric artifacts of language articulation [5], [7], [15]. If participants did not respond within 2000 ms, the picture disappeared. Afterwards, a blank screen lasting 1000 ms appeared and then the next trial started.

There were two types of trial sequences: switch and repeat trials. In repeat trials, the response language of two subsequent trials was the same (L1L1 or L2L2), whereas in switch trials, the response language of the current trial was different from the response language of the previous trial (L1L2 or L2L1). There were 5 blocks and 96 trials per block. Each block contained 24 repeat trials of L1L1 and L2L2, as well as 24 switch trials of L1L2 and L2L1, respectively. All stimuli were pseudo-randomly presented and participants practiced before the formal experiment. Reactions were recorded by PSTSR-BOX which was connected to a microphone.

### Electrophysiological recordings

Electrophysiological data were recorded from 64 Ag/AgCl electrodes placed according to the extended 10–20 positioning system (http://www.neuroscan.com/). The signal was recorded with 1 kHz sampling rate and referenced online to the right mastoid (M2). Impendences were kept <5 kΩ. Electroencephalographic activity was filtered online within a bandpass between 0.1 and 200 Hz and refiltered offline with a 30 Hz, low-pass, zero-phase shift digital filter. Eye blinks were mathematically corrected [27] and remaining artifacts were manually rejected. Continuous recordings were cut into epochs ranging from −200 to 1000 ms after the onset of each trial. Baseline correction was performed in reference to pre-stimulus activity (−200–0 ms) and individual averages were digitally re-referenced to a global average reference. Signals exceeding ±80 µV in any given epoch were automatically discarded.

### Behavioral data analysis

The naming latencies were analyzed, whereas accuracy scores were ignored due to the high accuracy (>95%).

The data from the first two trials of each block and naming latencies beyond *M±*3*SD* were excluded (14.08%), and only RTs of correct trials were analyzed. L1 switch costs were calculated by means of the difference between switch RTs under the condition of L2L1 (switching from L2 to L1) minus the condition of L1L1 (switching from L1 to L1) (i.e., L2L1–L1L1). The calculation method of L2 switch costs was analogous to L1 switch costs (L1L2–L2L2) [1], [8], [18], [28].

For naming latencies, a three-way repeated-measures ANOVA with the between-subject factor group (high vs. low inhibitory control group) and the two within-subject factors language (L1 vs. L2), and task (repeat vs. switch) was conducted on the RTs.

For switch costs of naming latencies, a two–way repeated measures ANOVA with the between–subject factor group (high vs. low inhibitory control) and the within-subject factor language switch costs (L1 vs. L2) was conducted.

### Event-related brain potential analysis

ERP components were defined based on the grand averages and analyzed in time–windows classically used to explore the N2 and LPC. As a result, repeated measures ANOVAs were performed on mean amplitudes in the following intervals: 260–380 ms for the N2, and 450–650 ms for the LPC. Topographical analyses were based on mean amplitudes measured over 64 electrodes distributed over the entire scalp. A Greenhouse–Geisser correction was performed where applicable. According to previous studies on language switching and based on visual inspection of the current data [5], [7], [8], [15], we focused the analysis of the N2 and LPC over three regions of interest (ROIs): frontal: F3, F1, Fz, F2, F4; fronto–central: FC3, FC1, FCZ, FC2, FC4; central: C3, C1, CZ, C2, C4.

We analyzed the difference between the high–IC and low–IC group regarding the ERP components N2 and LPC. The data from the first two trials of each block as well as naming errors and trials contaminated by artifacts were removed from the analyses (18.37%). A five–way repeated–measures ANOVA with the between–subject factor group (high vs. low inhibitory group) and the four within-subject factors language (L1 vs. L2), task (repeat vs. switch), hemisphere (left, midline and right), and brain region (frontal vs. fronto–central vs. central) was conducted on the mean amplitudes. Whenever a main effect of group, language, and task and/or an interaction containing one or more of these factors reached significance (*p*<.05) subsequent simple effects analyses were performed. Please note that a significant three–way interaction between group, language, and task will provide direct support for our assumption that inhibition could modulate language switch costs.

## Results

### Behavioral results

**Figure 1:**
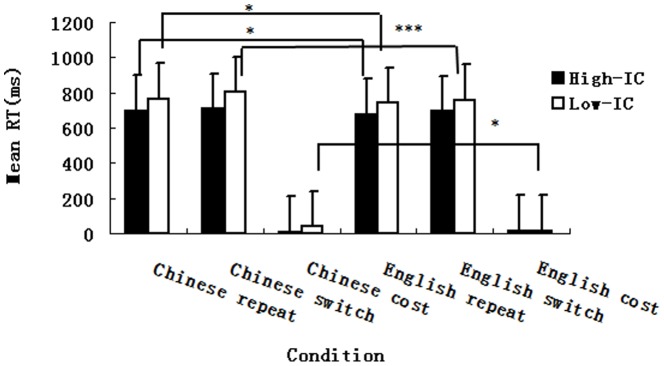
Mean latencies (ms), language switch costs, and standard errors for the high and low inhibitory control (IC) group during the EEG experiment.

Mean latencies and language switch costs for the high–IC and low–IC group are shown in Figure 1.

For naming latencies, the statistical analysis yielded main effects of language (*F*(1, 40) = 17.07, *MSE*  = 1529, *η^2^_P_* = .30, *p*<.001), and task (*F*(1, 40) = 22.75, *MSE*  = 758, *η^2^_P_* = .36, *p*<.001). The three–way interaction of group, language, and task also reached significance (*F*(1, 40) = 5.15, *MSE*  = 402, *η^2^_P_* = .11, *p*<.05). A simple effect analysis of the three–way interaction showed that L1 repeat RTs were longer than that of L2 repeat RTs for both high–IC and low–IC groups (*F*(1, 40) = 5.14, *p*<.05, *F*(1, 40) = 5.94, *p*<.05). Furthermore, L1 switch RTs were longer than that of L2 for the low-IC group (*F*(1, 40) = 18.41, *p*<.001), but they did not differ for the high–IC group (*F*(1, 40) = 1.87, *p*>.05).

For switch costs, the statistical analysis yielded a significant interaction between group and language switch costs (*F*(1, 40) = 5.15, *MSE*  = 803, *η^2^_P_* = .11, *p*<.05). A simple effect analysis of this interaction showed that the difference between L1 and L2 switch costs for high–IC group was not significant (*F*(1, 20) = .73, *p*>.05), indicating that language switch costs were symmetrical. On the other hand, L1 switch costs were larger than that of L2 for the low–IC group (*F*(1, 20) = 4.93, *p*<.05), indicating that language switch costs were asymmetrical.

### ERP results

**Figure 2:**
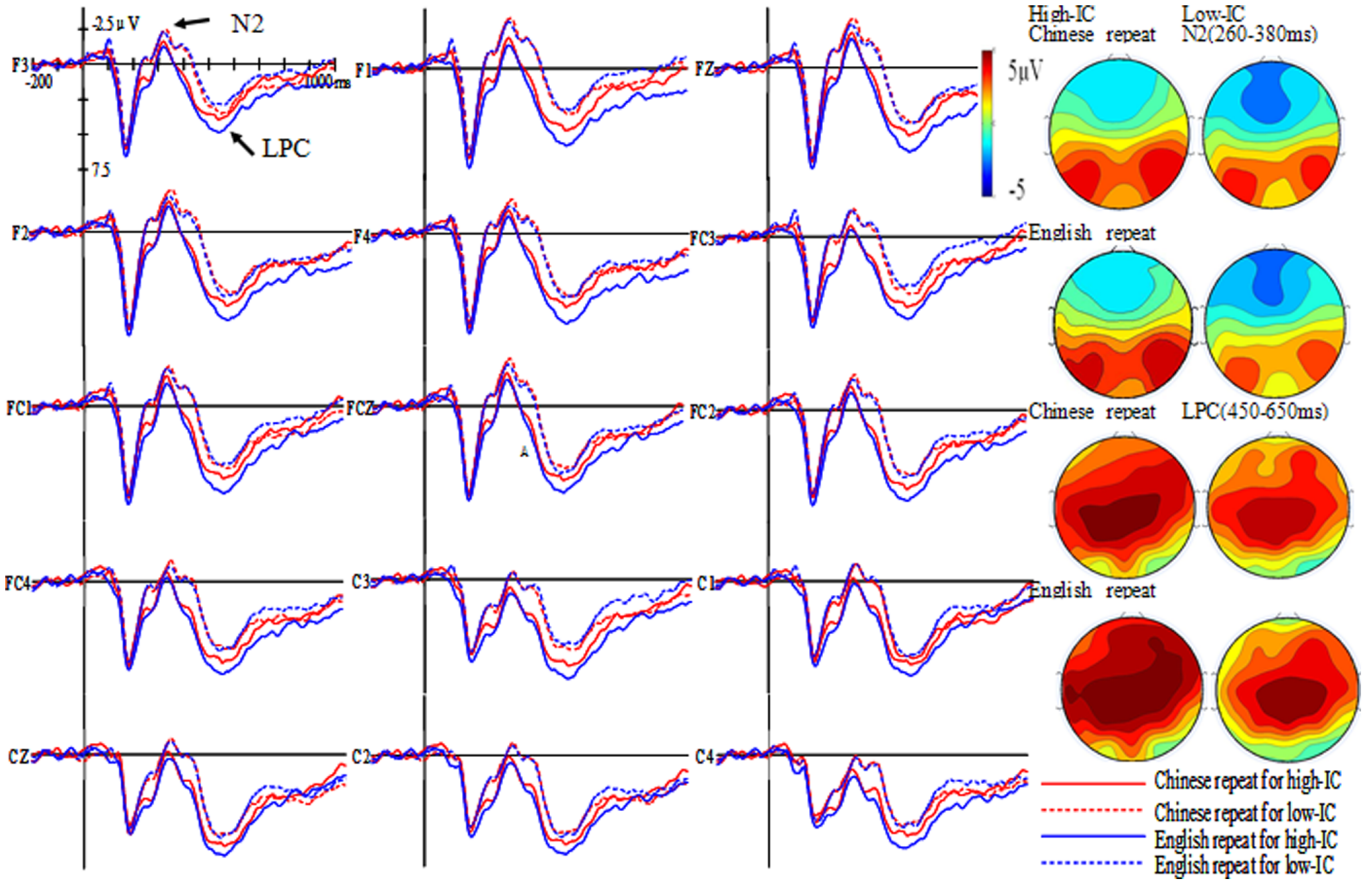
Grand average waveforms and topographic maps for L1 and L2 repeat trials for the high and low inhibitory control (IC) group.

**Figure 3:**
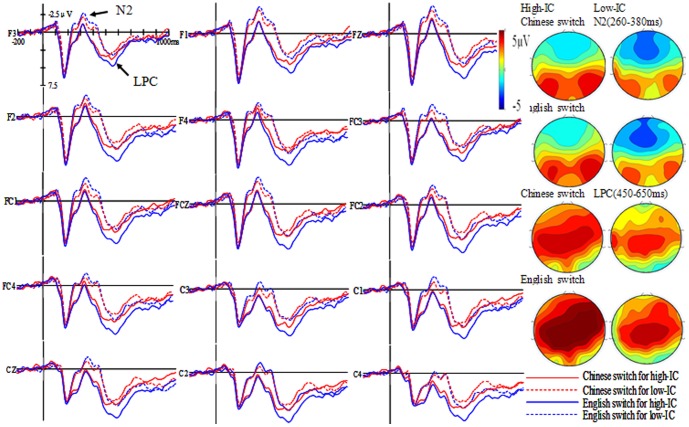
Grand average waveforms and topographic maps for L1 and L2 switch trials for the high and low inhibitory control (IC) group.

Grand average waveforms and topographic maps of L1 and L2 repeat and switch trials for both the high and low inhibitory control group are shown in Figure 2 and Figure 3.

#### N2 time window (260–380 ms)

The three-way interaction between group, language, and task failed to reach significance (*F*(1, 40) = .74, *MSE*  = 7.34, *η^2^_P_* = .02, *p>*.05), indicating that inhibition may not come into play during the language task schema competition phase. The three-way interaction between group, task and hemisphere reached significance (*F*(2, 80) = 2.62, *MSE*  = .73, *η^2^_P_* = .06, *p* = .09). A simple effect analysis of this three–way interaction showed that the amplitude in repeat trials did not differ across hemispheres neither for the high–IC group (*F*(2, 80) = .65, *p*>.05) nor for the low–IC group (*F*(2, 80) = 1.94, *p*>.05). Similarly, the amplitude of switch trials did not differ across hemispheres for the high–IC group (*F*(2, 80) = .69, *p*>.05), but the amplitude of switch trials at midline sites was larger than in the left and right hemisphere for the low–IC group (*F*(2, 80) = 5.11, *p*<.01). Furthermore, the three–way interaction between group, language, and hemisphere reached significance (*F*(2, 80) = 6.80, *MSE*  = .55, *η^2^_P_* = .15, *p*<.01). A simple effect analysis of the three-way interaction showed that, for the high–IC group, the amplitude was not significantly different across hemispheres neither for L1 trials (*F*(2, 80) = 1.03, *p*>.01) nor for L2 trials (*F*(2, 80) = .84, *p*>.01). However, for the low–IC group, the N2 amplitude at midline sites was larger than in the left and right hemisphere for L1 trials (*F*(2, 80) = 2.74, *p* = .07) and L2 trials (*F*(2, 80)  = 5.76, *p*<.01).

#### LPC time window (450–650 ms)

The three–way interaction between group, language, and task reached significance (*F*(1, 40) = 4.99, *MSE*  = 17.97, *η^2^_P_* = .11, *p*<.05), indicating that inhibition may play a key role during the lexical response selection phase. A simple effect analysis of this three–way interaction showed that the amplitude in L2 repeat trials was larger than that in L1 trials for the high–IC group (*F*(1, 40) = 4.10, *p* = .05), but no difference was present for the low–IC group (*F*(1, 40) = .00, *p*>.05); the amplitude in L2 switch trials was larger than that in L1 trails for the high–IC group (*F*(1, 40) = 10.94, *p*<.01), but again no difference was present for the low–IC group (*F*(1, 40) = .82, *p*>.05). The three–way interaction between group, task, and hemisphere also reached significance (*F*(2, 80) = 5.70, *MSE*  = .40, *η^2^_P_* = .13, *p*<.01). A simple effect analysis of this three–way interaction showed that the amplitude in repeat trials was not significantly different across hemispheres neither for the high–IC group (*F*(2, 80) = 2.18, *p*>.05) nor for the low–IC group (*F*(2, 80) = 2.83, *p*>.05); furthermore, the amplitude in switch trials was larger in the right hemisphere than in the left and at midline sites for the high–IC group (*F*(2, 80) = 5.47, *p*<.01), but not for the low–IC group (*F*(2, 80) = 2.12, *p*>.05). The two–way interaction between language and hemisphere reached significance (*F*(2, 80) = 8.31, *MSE*  = .49, *η^2^_P_* = .17, *p*<.01). A simple effect analysis of this two–way interaction showed that the amplitude of L1 trials was larger in the right and left hemispheres than at midline sites (*F*(2, 82) = 3.39, *p*<.05). A similar effect was presented also for L2 trials (*F*(2, 82) = 9.36, *p*<.001).

## Discussion

The present study aimed at exploring the impact of domain–general inhibitory control on language switching, and the underlying timing of inhibitory processes. The main finding of the behavioral results was that the high–IC group showed symmetrical language switch costs, whereas the low–IC group showed asymmetrical switch costs, indicating that domain–general inhibitory control seems to play an important role in modulating language switch costs (symmetrical or asymmetrical). The ERP results concerning the N2 component failed to show a significant interaction between group, language, and task, while the LPC revealed such a three–way interaction, indicating that inhibition may play a key role during the lexical response selection phase.

### Domain–general inhibitory control modulates language switching

In the present study, both asymmetrical and symmetrical language switch costs were observed as a function of inhibitory control for low–proficient Chinese–English bilinguals. As the IC model predicts [2], [4], [5], [28], participants exhibiting high domain–general inhibitory control are equipped with strong inhibitory control mechanisms during language switching, and this ability may offset the effect of low English proficiency on language switch costs. Specifically, when Chinese native speakers with high or low inhibitory control perform L2 repeat trials, Chinese as their L1 is easily activated consuming a large amount of cognitive resources to suppress it continuously. Since these trials necessitate a stronger inhibition of L1, once participants switch from L2 to L1, they need more time relieving this strong inhibition, which leads to larger L1 switch costs. High domain–general inhibitory control may offset the effect of a low L2 proficiency on language switch costs. Following this line of thinking, it seems plausible that the high–IC group with a strong ability of suppression elicited symmetrical language switch costs, while the low–IC group with a weak ability of suppression resulted in asymmetrical language switch costs. Thus, domain–general inhibitory control seems to play a similar role as inhibitory control occurring in a language–specific context.

In addition, our results are consistent with Linck and colleagues' study [3]. Their results showed that the stronger domain–general inhibitory control was, the better the performance of language switching, indicating that both the Simon task and language switching rely on common domain-general inhibitory control mechanisms. Recently, De Bruin et al. [2] also found that the right inferior frontal gyrus (rIFG) and the pre-supplementary motor area (pre–SMA) were both involved in switching to L2 and L3 and can be considered to be associated with domain-general inhibition. In sum, our findings are in line with other studies emphasizing the role of domain-general inhibitory control during language switching. However, our ERP study provides further fine-grained information on how domain-general inhibitory control impacts the time course of language switching. Specifically, we were interested in whether inhibition occurs during an early phase of language task schema competition or during a later phase of lexical response selection.

### N2 as a reflection of attentional control acting during the language task schema competition phase

In the present study, the N2 peaked around 320 ms after picture stimulus onset, which is in line with other studies [5], [7], [8], [9], [15]. According to the IC model, domain–general inhibitory control can modulate language switching, that is, high–IC participants are able to exhibit more inhibitory processes to suppress the interference from the non–target language, especially, when switching from L1 to L2. Thus, Chinese native speakers should inhibit their native language more strongly. Following this model we would have expected the high–IC group to display a larger N2 amplitude than the low–IC group in L2 switch trials than L1 trials. However, the results failed to show a significant interaction between group, language, and task indicating that inhibitory control may not come into play during the language task schema competition phase. Furthermore, the N2 amplitude of switch trials was larger at midline sites than in the left and right hemisphere for the low-IC group, and the amplitude of midline sites (i.e., FZ, FCZ, CZ) was larger than that of the left and right hemisphere for both L1 and L2 trials. These findings indicate that the N2 component was clearly distributed over midline brain areas. From previous studies we know that the right inferior frontal gyrus (rIFG) is the main area responsible for inhibitory control [2], [16]–[19]. Because in our experiment the distribution was fronto–centrally localized, we suggest that the N2 may not represent inhibitory control per se. Additionally, if inhibition is expected to occur at the lexical response selection phase, as proposed by the model, it should not occur in such an early time window (between 260–380 ms).

Martin and colleagues' study [15] found that the N2 component was larger for early bilinguals than for late bilinguals when early and late bilinguals had to name pictures in their L1 and L3. They proposed that the N2 component does not reflect inhibition per se but a mechanism involved in cognitive control. Verhoef and colleagues [9] also suggested that the role of N2 in language switching was associated with attentional control rather than inhibition. Studies investigating different domain-general cognitive tasks also provided evidences for the N2 reflecting attentional control, as they found a modulation of the N2 when focusing attention on targets and filtering out irrelevant stimuli was required [10]–[14]. Thus, we speculated that what we saw in this early time window was not inhibition but probably a reflection of an attentional control process in which the appropriate language (i.e, L1 or L2) is selected during the language task schema competition phase.

### LPC as a reflection of lexical inhibition acting during the lexical response selection phase

The high–IC group of the present study displayed a larger LPC amplitude for L2 repeat and switch trials than for L1 trials in the time window 450–650 ms. The low–IC group, on the contrary, did not show such a difference. These results were partly consistent with some previous studies. For example, Martin and colleagues [15] found a larger LPC in L1L2 early bilinguals than L1L3 early bilinguals during picture naming, as well as in switch than in non–switch trials when responses were given in L3 but not when they were given in L1. Jackson et al. [5] also found that both L1 and L2 showed sustained increase in LPC amplitude for switch trials compared to non-switch trials during digit naming.

In our study, the LPC occurred between 450 and 650 ms. According to the IC model, if the LPC represents inhibition during lexical selection, we would have predicted a larger LPC the more lexical selection inhibition is required. Indeed, our results fit to this assumption by revealing in high–IC participants a larger LPC for L2 switch and repeat trials than for L1 trials. Because the high–IC group possesses high inhibitory control ability, they have enough inhibition to suppress the non–target language in the late processing phase. Interestingly, our results for the high–IC group display a similar LPC for L2 repeat and switch trials than L1 ones, leading to symmetrical language switch costs, while the low–IC group did not, indicating that inhibition occurs during the lexical selection phase. Moreover, the results showed that the LPC amplitude in switch trials was larger in the right hemisphere (i.e., F2, F4, FC2, FC4, C2, C4) than in the left and at midline sites for the high-IC group, but not for the low–IC group. This result is in line with previous findings suggesting the right inferior frontal gyrus (rIFG) as the main area responsible for inhibitory control [2], [16]–[19]. Taken together, these results provide further evidence in support of the assumptions of the IC model in language switching.

In conclusion, the present study showed differential language switching patterns between participants with high and low inhibitory control abilities. These findings provide direct evidence that domain–general inhibitory control could be generalized to language switching, and that domain–general inhibitory control acts during the lexical response selection phase. Thus, our study provides important evidence with respect to two debated issues of the IC model. First, inhibition not only refers to domain–specific inhibition underlying the language network, but also involves domain–general inhibition. Second, our ERP study reveals explicit electrophysiological mechanisms on how domain–general inhibitory control impacts the time course of language switching, namely during the lexical response selection phase. Summarizing, the present study provides new and important electrophysiological evidence for the long–standing claim of the involvement of inhibition during language switching.

## Supporting Information

Table S1 **Demographic and language proficiency data.**
(XLS)

Table S2 **Language switching materials.**
(XLS)

Table S3 **Simon task latency data.**
(XLS)

Table S4 **Language switching latency data.**
(XLS)

Table S5 **N2 amplitude data.**
(XLS)

Table S6 **LPC amplitude data.**
(XLS)
